# Effects of nitrogen application rate on the photosynthetic pigment, leaf fluorescence characteristics, and yield of indica hybrid rice and their interrelations

**DOI:** 10.1038/s41598-021-86858-z

**Published:** 2021-04-05

**Authors:** Jinfeng Peng, Yuehua Feng, Xiaoke Wang, Jie Li, Guiling Xu, Somsana Phonenasay, Qiangxin Luo, Zhili Han, Wei Lu

**Affiliations:** 1 College of Agronomy, Guizhou University, Guiyang, 550025 China; 2 Key Laboratory of Plant Resource Conservation and Germplasm Innovation in Mountainous Region (Ministry of Education), Guizhou University, Guiyang, 550025 China

**Keywords:** Physiology, Agroecology

## Abstract

A field experiment employing the rice cultivars Qyou6 and Yixiangyou2115 as materials and different nitrogen application rates was conducted in Huangping County, Guizhou Province in 2019 to determine the effects of nitrogen application rate on photosynthetic pigments, leaf fluorescence characteristics, yield, and their interrelations in indica hybrid rice. The results showed that photosynthetic pigment contents generally increased with increasing nitrogen application rate. As the nitrogen rate increased, the maximal quantum yield of PSII (F_v_/F_m_), actual quantum yield of PSII (Φ_PSII_), and relative electron transfer rate at PSII (ETR) first decreased and then increased at the booting stage; F_v_/F_m_ and Φ_PSII_ decreased while ETR first increased and then decreased at the heading stage; nevertheless, F_v_/F_m_ and Φ_PSII_ first decreased and then increased but ETR was just the opposite at the maturity stage. Non-photochemical quenching coefficient (qN) and quantum yield of regulatory energy dissipation at PSII (Y(NPQ)) first increased and then decreased whereas quantum yield of non-regulatory energy dissipation at PSII (Y(NO)) first decreased and then increased at the booting, heading, and maturity stages with increasing nitrogen application rate. Photochemical quenching coefficient (qP) showed an increasing trend as the nitrogen rate increased in the range of 150–300 kg/ha at the heading and maturity stages. Photosynthetic pigments, leaf fluorescence characteristics, and yield and its components were significantly correlated. First, chlorophylls a and b were significantly negatively correlated with F_v_/F_m_ while significantly positively correlated with qP at the heading stage. Secondly, Carotenoids were significantly positively correlated with the effective panicle number (EPN) at the booting stage while significantly negatively correlated with the spikelets per panicle (SPP) at the heading stage. Chlorophyll a and carotenoids were significantly positively correlated with EPN but significantly negatively correlated with spikelet filling (SF) at the maturity stage. In addition, qP was significantly negatively correlated with EPN at the booting stage. At the heading stage, F_v_/F_m_ and Y(NO) were significantly negatively correlated with EPN and SPP, respectively, and F_v_/F_m_ and Φ_PSII_ were significantly positively related to SF. Moreover, qP was extremely significantly positively related to EPN whereas F_v_/F_m_ was extremely significantly negatively correlated with grain yield at the maturity stage. Appropriate nitrogen application rates can enhance photosynthetic pigment contents, improve the photochemical efficiency and proportion of the open part of the reaction center of PSII, and promote the quantum efficiency and self-protection ability of PSII, thereby increasing photosynthetic efficiency and yield. Under the conditions adopted in this experiment, a parabolic relationship was observed between the nitrogen application rate and grain yield. The regression analysis results showed that the best nitrogen application rate of indica hybrid rice is 168.16 kg ha^−1^ and the highest yield is 11,804.87 kg ha^−1^.

## Introduction

Nitrogen is an important factor for increasing rice yield. Among the many factors that could promote rice yield, the contribution of nitrogen fertilizer exceeds 40%^1^. Nitrogen supply during rice growth and development not only exerts evident regulatory effects on leaf photosynthetic characteristics, stalk and sheath storage output and transport, grain carbohydrate accumulation, and sinks source balance, but also has a close relationship with grain filling and yield^2,3^. Lin et al.^4^ found that long-term non-application of nitrogen causes soil nitrogen and fertility to decrease, which reduces crop yields, whereas application of nitrogen fertilizer increases the total nitrogen content of the soil; this study further found that the proper application of nitrogen has a significant effect on crop yield.

The supply of nutrients in the cultivation environment is directly related to the photosynthetic function of crop leaves^5^. The photosynthetic pigments of higher plants include chlorophyll and carotenoids^6^. Chlorophyll is an important pigment involved in the absorption, transmission, and transformation of light energy in photosynthesis^7^. Several studies have indicated that nitrogen nutrition plays an important role in the regulation of photosynthetic pigment synthesis in crop leaves, and that the level of nitrogen supply has a significant positive correlation with the chlorophyll content of crops^8,9^. Therefore, when the crop lacks nitrogen, its chlorophyll content decreases, its photosynthetic efficiency weakens, carbohydrate synthesis is blocked, and crop yield declines^10^.

Chlorophyll fluorescence emitted by plants is also closely related to various reaction processes in photosynthesis and may contain rich information about photosynthetic changes. Scholars at home and abroad have carried out considerable research on the effect of nitrogen application on the fluorescence characteristics and yield properties of crop leaves^11–15^. Nevertheless, different research conclusions on the relationship between nitrogen application and fluorescence characteristics of rice leaves have been reported. Xu et al.^13^, for example, demonstrated that, under the same irrigation mode, proper nitrogen treatment could increase F_v_/F_m_ in rice leaves. However, Ma et al.^16^ showed that F_v_/F_m_ in rice leaves increases with increasing nitrogen application under three nitrogen supply levels in field experiments.

Research on the relationships among rice leaf photosynthetic pigments, fluorescence characteristics, and yield properties is limited. For instance, Liu et al.^17^ only used an indica japonica hybrid as an experimental material to study the relationship between chlorophyll content and rice yield components. Yan et al.^18^ only studied the relationship between chlorophyll content and several fluorescence parameters (F_v_/F_m_, F_v_/F_o_, ETR) after the full spreading of rice flag leaves. Xu et al. ^13^ studied the relationship between F_v_/F_o_, F_v_/F_m_, and rice yield at the middle tillering and heading stages under the condition of pool planting only. Guo et al.^19^ explored the relationships among a limited number of fluorescence parameters, including F_v_/F_m_, Φ_PSII_, qP, and qN, and yield traits after rice heading.

In the present study, photosynthetic pigments, leaf fluorescence characteristics, yield and its components were determined in two indica hybrid rice cultivars under five nitrogen application rates in 2019. The purpose of this study is to explore the effects of nitrogen application rate on photosynthetic pigments, leaf fluorescence characteristics, yield, and their interrelations in indica hybrid rice.

## Results

### Photosynthetic pigments in rice leaf blades

**Figure 1:**
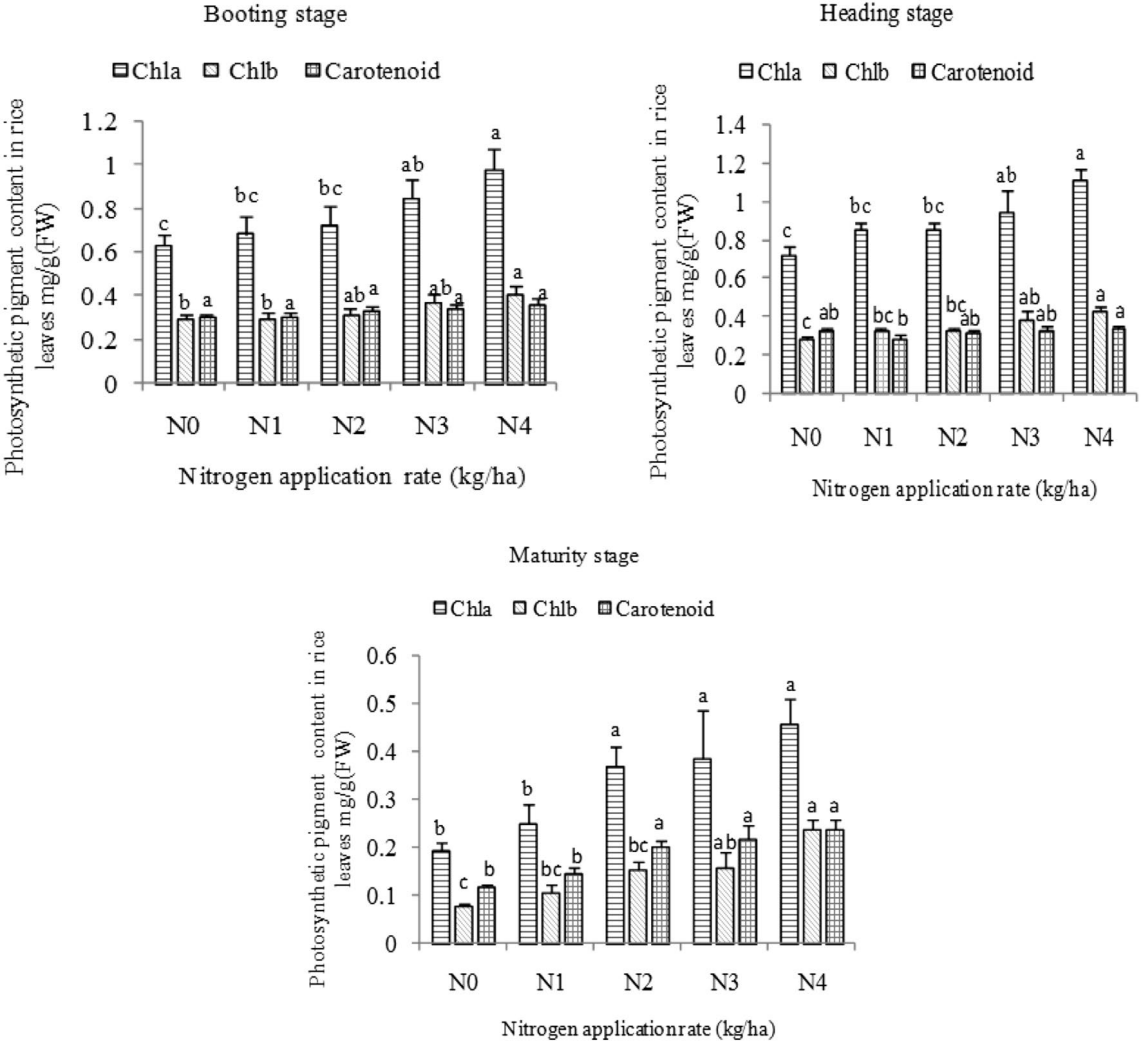
Effects of different nitrogen application rates on photosynthetic pigments in rice leaves. *Note* N0:0 kg ha^-1^; N1:75 kg ha^-1^; N2:150 kg ha^-1^; N3: 225 kg ha^-1^; N4:300 kg ha^-1^; Chla, chlorophyll (**a**); Chlb, chlorophyll (**b**); significant differences between rice varieties and nitrogen treatments (*P* < 0.05) are indicated by lowercase letters; vertical bars represent SE (n = 3). The data in the figure is the average value of the two cultivars.

The contents of chlorophylls a, and b and carotenoids showed an upward trend with increasing nitrogen application rate. Pigments in the N4 treatment were significantly higher than those in the N1 treatment at the heading and maturity stages (Fig. 1).

### Photochemical efficiency of rice leaf blades

Photochemical efficiency is calculated from to the number of photons of the product obtained by the effective photochemical reaction divided by the total quantum number of light absorbed. F_v_/F_m_ represents the maximum photosynthetic quantum yield of the reaction center of PSII and reflects the internal light energy conversion efficiency or maximum light energy conversion efficiency of PSII; this characteristic may indicate the photosynthetic potential of crops^20^. Φ_PSII_ represents the actual photosynthetic quantum yield of PSII and reflects the actual primary light energy capture efficiency of the reaction center of PSII when it is partially closed^21^. ETR reflects the actual electron transfer rate of PSII^21^.

**Figure 2:**
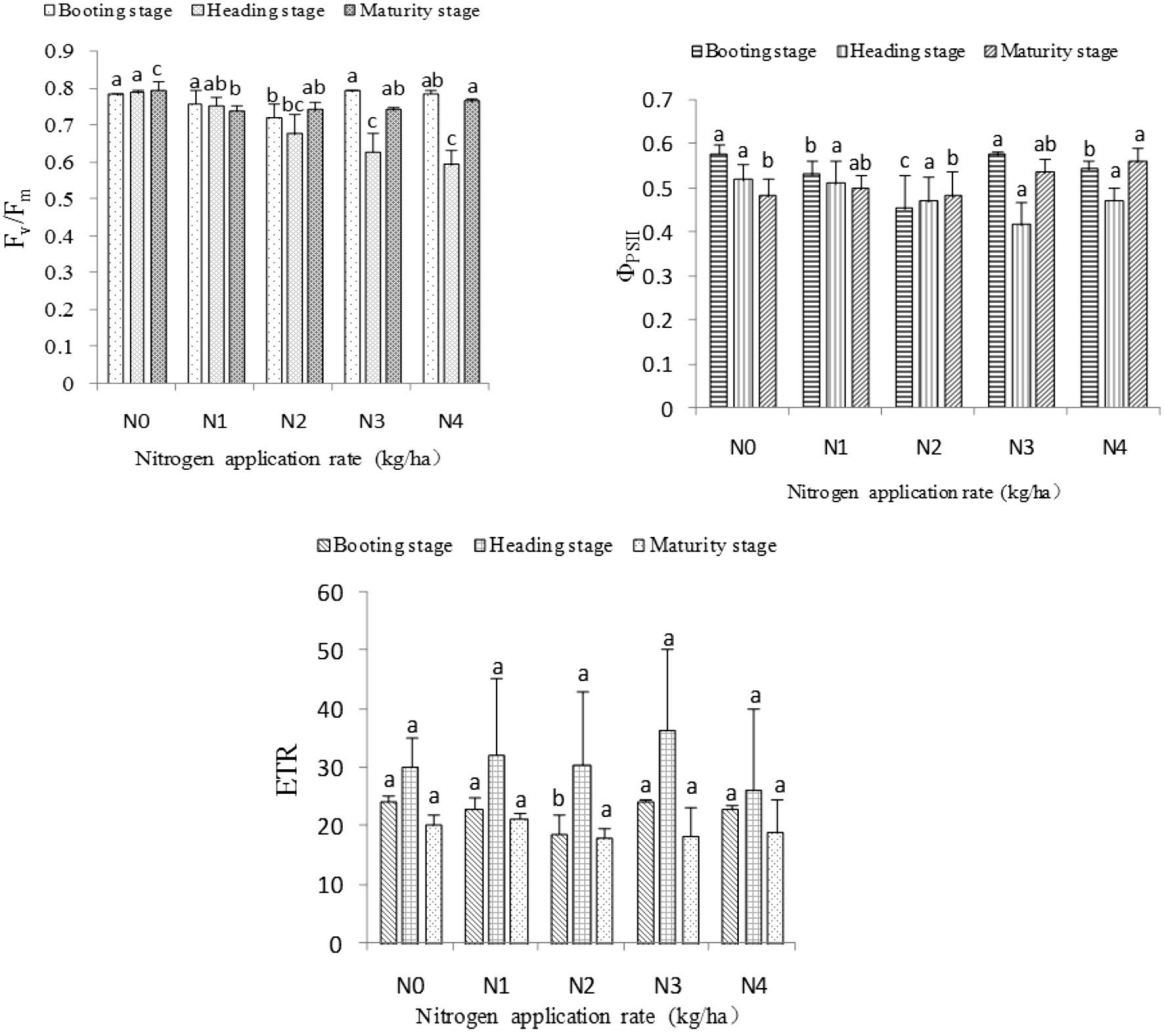
Effects of different nitrogen application rates on the photochemical efficiency of rice leaves.

As the nitrogen application rate increased, F_v_/F_m_ first decreased and then increased at the booting and maturity stages but decreased at the heading stage. F_v_/F_m_ in the N3 treatment was the highest and significantly higher than that in the N2 treatment at the booting stage. Moreover, F_v_/F_m_ in the N0 treatment was the highest and significantly higher than those in the N2, N3, and N4 treatments at the heading and maturity stages (Fig. 2).

As the nitrogen application rate increased, Φ_PSII_ first decreased and then increased at the booting stage, decreased at the heading stage, and then increased at the maturity stage. Compared with those of other treatments, Φ_PSII_ in the N0 treatment was significantly higher than those in the N1, N2, and N4 treatments at the booting stage. Among the treatments studied, the N0 treatment showed the highest Φ_PSII_ at the heading stage, but no significant difference was observed among the five nitrogen application treatments. Φ_PSII_ in the N4 treatment was the highest and significantly higher than that in the N0 and N2 treatments at the maturity stage (Fig. 2).

ETR showed a downward trend followed by an upward trend at the booting stage. However, at the heading and maturity stages, ETR first increased and then decreased. The highest ETR were observed in the N3 treatment at the heading stage and in the N1 treatment at the maturity stage, although differences between the treatments were not significant. ETR in the N0 treatment was highest and significantly higher than that in the N2 treatment at the booting stage (Fig. 2).

### Fluorescence quenching coefficient of rice leaf blades

Fluorescence quenching may be categorized as photochemical quenching and non-photochemical quenching. Photochemical quenching is represented by qP, which reflects the degree of reaction center opening in the redox state of the original electron acceptor QA of PSII^21^, while non-photochemical quenching is represented by qN, which reflects the part of light energy absorbed by pigments of the PSII antenna and dissipated in the form of heat instead of being used for photosynthetic electron transfer^22^.

**Figure 3:**
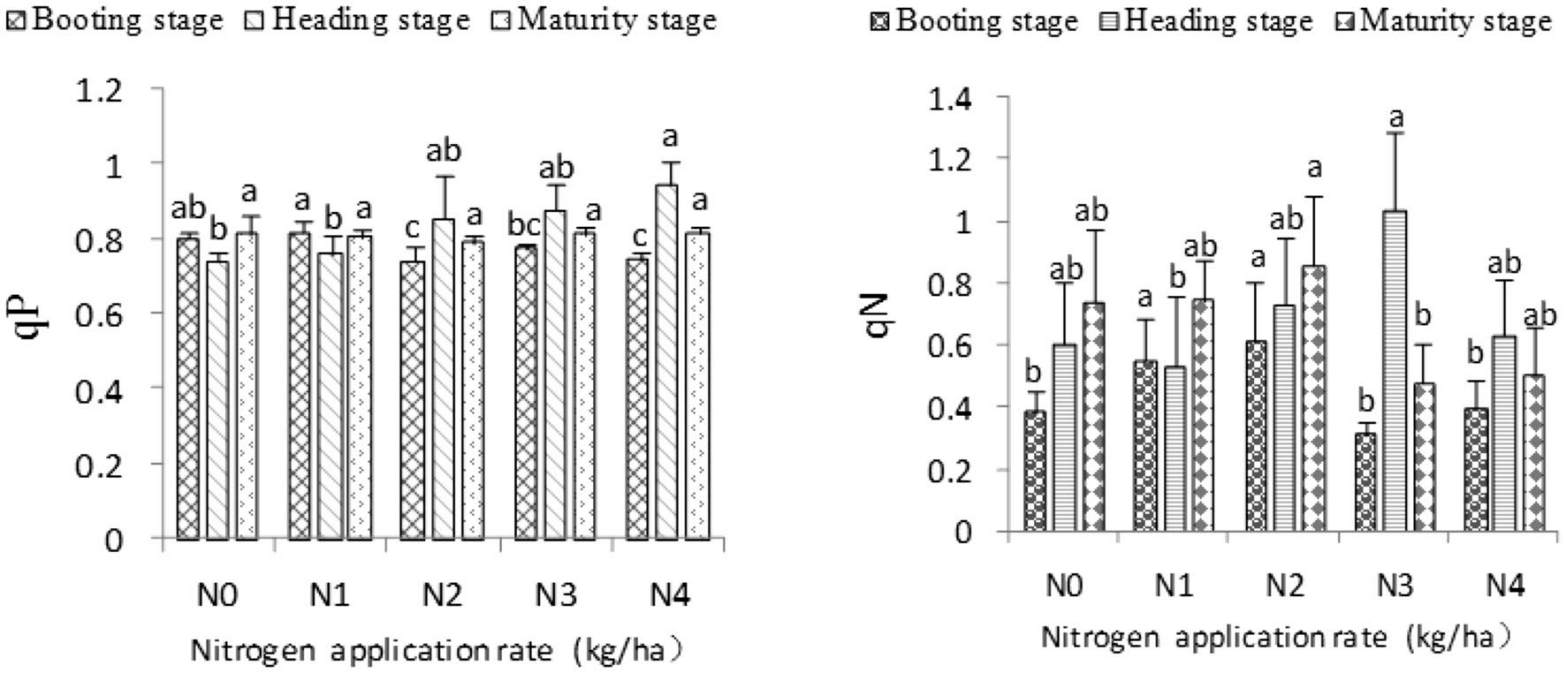
Effects of different nitrogen application rates on the fluorescence quenching coefficients of rice leaves.

Figure 3 demonstrates that the effect of nitrogen application rate on qP is not evident at the booting stage. However, qP increased at the heading stage, and first decreased and then increased at the maturity stage with increasing nitrogen application rate. Among the qPs obtained, that inthe qPs obtained, that in the N4 treatment was the highest. The qP in N4 was also significantly higher than that inin N1 and N2 at the heading stage. Similarly, the qP in the N4 treatment was the highest at the maturity stage, but no significant difference was found among treatments. qN first increased and then decreased at the booting, heading, and maturity stages with increasing nitrogen rate. Among the qNs obtained, that in the N2 treatment was the highest and significantly higher than that in N3 at the booting and maturity stages. Moreover, among the qNs obtained, the qN in the N3 treatment was the highest and significantly higher than that in the N1 treatment in the heading stage.

### Non-photochemical quenching quantum yield of rice leaf blades

Y(NPQ) refers to the quantum yield of regulatory energy dissipation at PSII, which is an important index of light protection^20^, while Y(NO) refers to the quantum yield of non-regulatory energy dissipation at PSII, which is an important index of light damage^20^.

**Figure 4:**
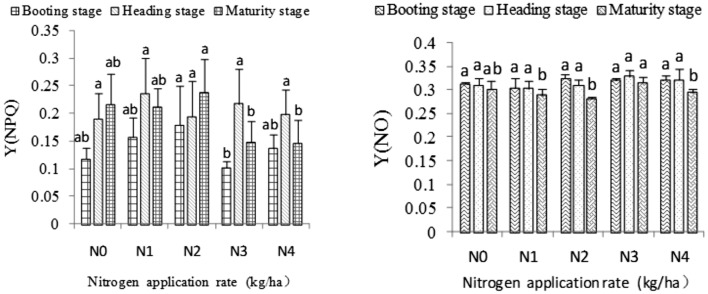
Effects of different nitrogen application rates on the quantum yield of non-photochemical quenching.

Y(NPQ) first increased and then decreased at the booting, heading, and maturity stages with increasing nitrogen application rate. The Y(NPQ) in the N2 treatment was the highest among the results obtained and significantly higher than that in the N3 treatment at the booting and maturity stages. However, at the heading stage, the highest Y(NPQ) was observed in the N1 treatment, although no significant difference occurred between the five nitrogen application rates. Y(NO) first decreased and then increased at the booting, heading, and maturity stages as the nitrogen rate increased (Fig. 4). The Y(NO) values in the N2 and N3 treatments were highest at the booting and heading stages, respectively, and no significant difference was observed among all N treatments. The Y(NO) in the N3 treatment was significantly higher than those in the N1, N2, and N4 treatments at the maturity stage (Fig. 4).

### Yield and its components

The grain yield first increased and then decreased with increasing nitrogen application rate (Table 1), and the grain yield of the N2 treatment was the highest and significantly higher than those of the N0 and N4 treatments. The regression equation between grain yield (*y*) and nitrogen application rate (*x*) was y =  − 0.0413x^2^ + 13.89x + 10,637 (R^2^ = 0.9121, *P* < 0.01). The best nitrogen application rate of rice was 168.16 kg ha^−1^, and the highest yield was 11,804.87 kg ha^−1^. The effective panicle number (EPN), thousand-grain weight (TGW), and spikelet filling (SF) first decreased and then increased with increasing nitrogen application rate. In particular, the EPN of the N4 treatment was the highest and significantly higher than that of the N1 treatment. The TGW and SF of the N0 treatment were the highest and significantly higher than those of the N2, N3, and N4 treatments. The spikelets per panicle (SPP) first increased and then decreased with increasing nitrogen application rate. Here, the SPP of the N2 treatment was the highest, although no significant difference was observed among all treatments (Table 1).

**Table 1:** Yield and its component under different treatments in rice.

Treatment	Effective panicle number (m^−2^)	Spikelets per panicle	Thousand-grain weight (g)	Spikelet filling (%)	Grain yield (kg ha^−1^)
N0	194.91 ab	208.20 a	34.50 a	86.14 a	10,569 c
N1	188.43 b	215.55 a	33.98 b	83.81 ab	11,553 ab
N2	205.56 ab	211.39 a	33.82 bc	82.32 b	11,882 a
N3	206.48 ab	205.10 a	33.56 c	80.88 b	11,446 ab
N4	215.28 a	204.42 a	33.79 bc	82.01 b	11,187 b
V1	205.37 a	237.79 a	31.31 b	79.73 b	12,445 a
V2	198.89 a	180.08 b	36.55 a	86.33 a	10,210 b
V	0.68	136.35**	1836.91**	22.80*	99.94**
N	1.43	0.70	11.98**	3.22*	6.64**
V*N	0.12	0.63	3.76*	1.59	5.62**

*Note* Significant differences between rice varieties and nitrogen treatments (*P* < 0.05) are indicated by lowercase letters, and * and ** indicate significant effects (*P* < 0.05) and extremely significant effects (*P* < 0.01), respectively. The data in the table is the average value of the two cultivars. The same as below.

Between the two rice cultivars studied, the EPN, SPP, and grain yield of V1 were higher than those of V2. However, the TGW and SF of the former cultivar were lower than those of the latter. In particular, the SPP and grain yield of V1 were significantly higher than those of V2 (Table 1).

The results of variance analysis showed that differences in the SPP, TGW, and grain yield between rice varieties were extremely significant, and SF displayed significantly different. Significant differences in TGW, SF, and grain yield were observed among the nitrogen application rates. The interactions of these characteristics between varieties and nitrogen application rates were not significant except for TGW and grain yield (Table 1).

### Relationship between photosynthetic pigments and fluorescence parameters in rice leaf blades

At the booting stage, photosynthetic pigments in leaf blades were negatively correlated with Y(NPQ), qP, and qN but positively correlated with Fv/Fm and Y(NO); however, the correlations observed did not reach significant levels (*P* > 0.05). At the heading stage, photosynthetic pigments in leaf blades were negatively correlated with F_v_/F_m_, Φ_PSII_, and ETR but positively correlated with Y(NO), qP, and qN. Chlorophylls a and b were significantly correlated with F_v_/F_m_, qP. At the maturity stage, leaf photosynthetic pigments were negatively correlated with F_v_/F_m_, ETR, Y(NPQ), qP, and qN but positively correlated with Φ_PSII;_ however, the correlations found did not reach significant levels (*P* > 0.05) (Table 2).

**Table 2:** Relationship between photosynthetic pigments and fluorescence parameters in rice leaves.

Growth period	Item	F_v_/F_m_	Φ_PSII_	qP	qN	Y(NPQ)	Y(NO)	ETR
Booting stage	Chla	0.391	0.089	− 0.623	− 0.398	− 0.205	0.541	0.044
	Chlb	0.519	0.231	− 0.579	− 0.547	− 0.364	0.547	0.171
	Carotenoid content	0.252	− 0.049	− 0.795	− 0.344	− 0.145	0.749	− 0.126
Heading stage	Chla	− 0.912*	− 0.533	0.914*	0.227	0.073	0.648	− 0.307
	Chlb	− 0.928*	− 0.621	0.907*	0.342	0.126	0.732	− 0.181
	Carotenoid content	− 0.509	− 0.347	0.566	0.340	− 0.741	0.693	− 0.344
Maturity stage	Chla	− 0.405	0.763	− 0.014	− 0.558	− 0.632	− 0.010	− 0.720
	Chlb	− 0.241	0.821	− 0.152	− 0.581	− 0.662	− 0.055	− 0.567
	Carotenoid content	− 0.450	0.768	− 0.014	− 0.587	− 0.655	0.060	− 0.735

*Note* Chla, chlorophyll a; Chlb, chlorophyll b.

### Relationships among rice yield and its components, photosynthetic pigments, and fluorescence parameters

At the booting stage, carotenoids had a significant positive correlation with EPN. At the heading stage, carotenoids had a significant negative correlation with SPP. At the maturity stage, chlorophylls a and b and carotenoids had significant positive correlations with EPN. However, chlorophyll a and carotenoids had a significant negative correlation with SF (Table 3).

**Table 3:** Relationship between rice yield and its components, photosynthetic pigments, and fluorescence parameters.

Growth period	Item	Photosynthetic pigment				Fluorescence parameter						
		chla	chlb	Carotenoid content		F_v_/F_m_	Φ_PSII_	qP	qN	Y(NPQ)	Y(NO)	ETR
Jointing stage	GY	0.163	0.01	0.232		− 0.696	− 0.761	− 0.389	0.626	0.667	0.297	− 0.72
	EPN	0.871	0.871	0.964**		0.177	− 0.12	− 0.892*	− 0.31	− 0.102	0.863	− 0.223
	SPP	− 0.702	− 0.807	− 0.746		− 0.683	− 0.481	0.491	0.813	0.674	− 0.61	− 0.363
	SF	− 0.759	− 0.714	− 0.803		0.014	0.231	0.105	0.618	− 0.008	− 0.619	0.263
	TGW	− 0.705	− 0.649	− 0.728		0.052	0.245	0.525	0.042	− 0.05	− 0.515	0.257
Heading stage	GY	0.249	0.24	− 0.493		− 0.378	− 0.441	0.332	0.259	0.389	− 0.039	0.271
	EPN	0.787	0.778	0.764		− 0.913*	− 0.648	0.953**	0.444	− 0.479	0.751	− 0.331
	SPP	− 0.479	− 0.594	− 0.942**		0.652	0.56	− 0.657	− 0.538	0.482	− 0.884*	0.122
	SF	− 0.742	− 0.787	− 0.161		0.895*	0.901*	− 0.825	− 0.693	− 0.223	− 0.699	− 0.27
	TGW	− 0.711	− 0.757	− 0.028		0.84	0.865	− 0.757	− 0.655	− 0.35	− 0.627	− 0.327
Maturity stage	GY	0.459	0.301	0.483		− 0.935**	− 0.008	0.771	0.207	0.149	− 0.449	− 0.436
	EPN	0.915*	0.911*	0.899*		− 0.036	0.7	0.176	− 0.557	− 0.615	0.11	− 0.779
	SPP	− 0.564	− 0.586	− 0.567		− 0.361	− 0.696	− 0.66	0.78	0.775	− 0.629	0.518
	SF	− 0.881*	− 0.75	− 0.917*		0.719	− 0.651	0.404	0.542	0.589	− 0.158	0.704
	TGW	− 0.827	− 0.692	− 0.867		0.8	− 0.617	0.17	0.506	0.551	0.128	0.62

*Note* GY, grain yield; EPN, effective panicle number; SPP, spikelets per panicle; SF, seed setting rate; TGW, thousand-grain weight.

At the booting stage, qP was negatively correlated with EPN at the 5% significance level, and the correlation coefficient was − 0.892. At the heading stage, F_v_/F_m_ and Y(NO) were negatively correlated with EPN and SPP (*P* < 0.05) with correlation coefficients of − 0.913 and − 0.884, respectively, and F_v_/F_m_ and Φ_PSII_ were positively correlated with SF (*P* < 0.05) with correlation coefficients of 0.895 and 0.901, respectively. Moreover, qP was positively correlated with EPN(*P* < 0.05) with a correlation coefficient of 0.953. At the maturity stage, F_v_/F_m_ was negatively correlated with grain yield (*P* < 0.05) with a correlation coefficient of − 0.935 (Table 3).

## Discussion

Chlorophyll is the main participant and carrier of energy conversion in photosynthesis^23^. As important photosynthetic pigments, chlorophyll and carotenoids, which participate in the absorption, transmission, and distribution of photosynthetic light energy, play a key role in plant growth and development^24^. This study showed that chlorophyll a and b in rice leaves at the booting, heading, and maturity stages increase with increasing nitrogen application rate, thus indicating that nitrogen deficiency could lead to a decrease in chlorophyll content. This finding is consistent with the research of Gu et al.^10^. Zhang et al.^8^ reported that increased nitrogen application causes an evident increase in carotenoid content in wheat, similar to the results of the present study. This study also indicated that carotenoid increases with the increasing nitrogen application rate at the booting, heading (except for the N0 treatment), and maturity stages. Such findings indicate that nitrogen could significantly improve the content of photosynthetic pigments in leaves^25^ and, thus, has positive significance for the absorption of light energy by leaves and increase in photosynthetic rate^26^.

Nitrogen is an important component of chlorophyll and protein in plants. A lack of nitrogen can reduce the absorption of light energy by plants and the activity of the PSII reaction center^27^. Liu et al.^28^ reported that proper nitrogen application rates could improve the PSII activity, photochemical efficiency, and proportion of the open part of the PSII reaction center of rice leaves. These features help plants use captured light energy effectively during photosynthesis and promote the quantum efficiency and photosynthetic rate of PSII. Liu Qifeng's research is similar to the conclusion of this study; different from the results of this study was that the F_v_/F_m_ and Φ_PSII_ first increased and then decreased with increasing nitrogen application rate within the range of 150–300 kg ha^−1^ at the booting stage. When the nitrogen application rate was 225 kg ha^−1^, reaching the highest value, which also shows that the proper nitrogen application rate can enhance the photochemical efficiency of rice leaves. The potential active center of PSII could be damaged by excessively low or high nitrogen levels, thereby inhibiting the original reaction process of photosynthesis, negatively affecting the transfer of photosynthetic electrons from the PSII reaction center to the sink source, and resulting in reductions in F_v_/F_m_ and Φ_PSII_^29^.

In the present work, as nitrogen application rate increased, ETR at the heading and maturity stages first increased and then decreased, which indicates that the appropriate nitrogen application rate could improve the opening degree of the PSII reaction center of rice functional leaves and increase their light energy utilization rate. This mechanism may be related to the catalytic effect of nitrogen fertilizer on the activity of light-activated enzymes in leaves, which enhances the energy capture efficiency of the PSII reaction center^30^, and is consistent with the results of Zhang et al.^31^.

The present study found that qP increases gradually as the nitrogen rate increases within the range of 150–300 kg ha^−1^ at the heading and maturity stages. This result indicates that an appropriate increase in nitrogen application could increase the proportion of the open part of the PSII reaction center, which is beneficial to charge separation in the PSII reaction center. Charge separation enhances the flow of electrons from the PSII oxidation side to the PSII reaction center, thereby improving the PSII electron transfer capability and ultimately contributing to the increase in PSII quantum yield, which is consistent with the research of Ma Jifeng et al.^16^. Zhang et al. reported^31^ that qN under high-nitrogen treatment is greater than that under low nitrogen treatment, and surmised that the average opening degree of the PSII reaction center of rice leaves in under high nitrogen treatment was higher than that under low nitrogen treatment. The high electron transfer efficiency and heat dissipation ability of leaves under high nitrogen levels could avoid damage to the photosynthetic mechanism caused by excess light energy and improve their photosynthetic efficiency. This finding, however, is different from the results of the present study. The present results showed that qN first increases and then decreases with increasing nitrogen application rate within the range of 0–300 kg ha^−1^ at the booting, heading, and maturity stages. The reasons behind the differences observed must be further analyzed.

The light quantum absorbed by the PSII reaction center is mainly converted into energy Φ_PSII_, PSII regulated energy dissipation Y(NPQ) and non-regulated energy dissipation Y(NO) by photochemistry^32^, among which Y(NPQ) and Y(NO) represent the most important indices of light protection and light damage, respectively. Long et al.^29^ noted that the NPQ of low nitrogen treatment is higher than that of high nitrogen treatment 20 days after full heading, which indicates that high-nitrogen treated plants have poor self-protection ability in the later stages of rice growth, consistent with the results of the present study. This study found that Y(NPQ) first increases and then decreases at the three growth stages of rice with increasing nitrogen application. By contrast, Y(NO) showed the opposite trend. These results demonstrate that the appropriate amount of nitrogen application is conducive to enhance the self-protection ability of plants.

This study noted that SPP and grain yield first increase and then decrease with increasing nitrogen application, thus indicating that an appropriate amount of nitrogen application can promote rice grain yield. As the amount of nitrogen application exceeds a certain level, increases in crop yield, consistent with the results of Gao et al.^33^_;_ nmay be observed. EPN, TGW, and SF first decreased and then increased in the present study, which is ininconsistent with the results of Jiang et al.^34^. The difference found may be related to the variations in basic soil fertility.

Yan et al.^18^ showed that a significant or extremely significant positive correlation may be observed between chlorophyll a and b contents and F_v_/F_m_ and ETR during rice flag leaf senescence, which contrasts the findings of the present study. The present work found that, at the heading stage, chlorophyll a and b are negatively correlated with F_v_/F_m_ and ETR, among which, the former F_v_/F_m_ was significant while the latter chlorop ETR was not. These observed differences may be related to the research conditions and subjects. The relationship between most of the fluorescence parameters and contents of chlorophyll a and b in the present study did not show statistical significance, similar to the work of Xu et al.^35^.

Liu et al.^17^ reported that chlorophyll content is positively correlated with SPP at the 5% significance level and negatively correlated with SF and TGW at the 1% or 5% significance levels at the heading stage. Moreover, chlorophyll content was negatively correlated with SF at the 1% significance level and positively correlated with TGW at the 5% significance level at the maturity stage, which is different from the results of the present study. The results of the present study showed that chlorophyll content is negatively correlated with SPP, SF, and TGW at the heading stage, but the differences found were not significant. Chlorophyll content was negatively correlated with SF at the maturity stage, but only the relationship between chlorophyll a content and SF showed statistical significance. Chlorophyll content was negatively correlated with TGW, but the differences found were not significant. Moreover, a positive correlation between chlorophyll content and grain yield was observed at the heading and maturity stages, but the differences found were not significant. This finding contrasts the results of Zou et al.^36^, who noted a significant or extremely significant positive correlation between chlorophyll content and grain yield after full heading. Differences among these results may be due to the different materials and conditions used in the experiments.

The present study found that F_v_/F_m_ and Y(NO) are significantly negatively related to EPN and SPP, respectively, and that F_v_/F_m_ and Φ_PSII_ are significantly positively related to SF, and qP was positively correlated with EPN at the 1% significance level at the heading stage, but no significant correlation was observed between the fluorescence parameters and yield components at the maturity stage, which differs from the results of Guo et al.^19^. Guo et al. showed that no significant relationship could be observed between fluorescence parameters and yield components at the heading stage; however, at the maturity stage, F_v_/F_m_, Φ_PSII_, qP, and qN in rice leaves were significantly or extremely significantly positively correlated with SF and TGW. This difference may be due to the different materials and conditions used in the experiments. Xu et al.^13^ found that F_v_/F_m_ is significantly positively correlated with grain yield at the heading stage. Jiang et al.^12^ reported that Φ_PSII_ and qP are significantly positively correlated with grain yield at the heading stage, which is inconsistent with this study. The present study found that grain yield is negatively correlated with F_v_/F_m_ and Φ_PSII_ and positively correlated with qP at the heading stage, although the differences observed did not reach significant levels. In addition, only F_v_/F_m_ was significantly negatively correlated with grain yield at the maturity stage. These differences and their causes need further verification and analysis.

## Conclusions

Moderate fertilizer application can ensure high photosynthetic pigment content, enhance the ability of light energy capture, improve the photochemical efficiency and proportion of the open part of the reaction center of PSII, and promote the quantum efficiency and self-protection ability of PSII. These effects help avoid damage caused by excess light energy to the photosynthetic mechanism and promote high efficiency and yield. Significant correlations were observed among photosynthetic pigments, fluorescence characteristics, and yield and its components. The most suitable application amount of nitrogen fertilizer was 150 kg ha^−1^, and the yield of rice was 11,882 kg ha^−1^. The regression equation we obtained revealed that the best nitrogen rate was 168.16 kg ha^−1^ and the highest yield was 11,804.87 kg ha^−1^.

## Material and methods

### The collection of rice plant materials

In this study, field studies on rice cultivation, including the collection of rice plant materials, are in line with relevant institutional, national and international guidelines and legislation.

### Experimental site

The experiment was conducted in Zhaibi Village, Jiuzhou Town, Huangping County, Guizhou Province, China (26° 59′ 44.59″ N, 107° 43′ 58.90″ E), in 2019. The planting area had a subtropical humid climate, an altitude of 698 m, and an annual average temperature of 15.7 °C .The frost-free period and average annual rainfall were 268 days and 1200 mm, respectively.

### Soil properties and plant materials

The soil properties of the experimental field were as follows: pH of 5.02, organic matter of 18.38 g kg^−1^, alkali hydrolyzed nitrogen of 209. 50 mg kg^−1^, available phosphorus of 4.56 mg kg^−1^, available potassium of 65.73 mg kg^−1^, total nitrogen of 2. 62 g kg^−1^, total phosphorus of 0.29 g kg^−1^, and total potassium of 12.31 g kg^−1^. Two rice cultivars, Qyou6 and Yixiangyou2115, were employed in this study. Qyou6 was bred by the Chongqing Seed Company, and Yixiangyou2115 was selected by the Yibin Academy of Agricultural Sciences and Sichuan Agricultural University. The leaf color of the former was darker than that of the latter.

### Experimental details

A split-plot design with three replicates was adopted in this experiment. Two cultivars (Qyou 6 [V1] and Yixiangyou 2115 [V2]) were assigned to the main plots, and four nitrogen rates (0 [N0], 75 [N1], 150 [N2], 225 [N3], and 300 kg ha^-1^ [N4]) were assigned to the subplots. The size o f each subplot was 25.9 m^2^. Nitrogen fertilizer was split applied as follows: 35% basal, 20% at 7 days after transplanting, 30% at the panicle initiation stage, and 15% at the booting stage. Moreover, 96 kg P_2_O_5_ ha^-1^ and 67.5 kg K_2_O ha^-1^ were applied as basal fertilizers before transplanting for all treatments, and 67.5 kg K_2_O ha^-1^ was applied at the panicle initiation stage. Urea, superphosphate, and potassium chloride were used as nitrogen, phosphorus, and potassium fertilizers, respectively. A ridge covering measuring 30 cm in height and 20 cm in width was constructed around the subplots. This covering was pressed up to 30 cm underground to prevent the infiltration of water and fertilizer. A 50 cm-long walkway was established between replicates for field operation and investigation. The rice seedlings were raised on 18 April and transplanted on May 27 with a row spacing of 30 cm × 20 cm. One seedling was planted per hole. In the field, shallow water was maintained at the greening stage, and wet irrigation was carried out at the tillering stage. The water was cut off naturally to dry the field when the seedling number reached 85% of the expected panicle number. After jointing, water was irrigated into the field, and shallow water was maintained until the heading stage. Intermittent irrigation was carried out during the filling and ripening periods, and the water was cut off and dried 10 days before harvest. Diseases and pests in rice were controlled in a timely manner.

### Plant sampling and parameter determination

#### Determination of photosynthetic pigment content

Samples were collected at the jointing (24 July), heading (12 August), and maturity (14 September) stages. Leaves aged 1.5 were collected at the jointing stage, and flag leaves were collected at the heading and maturity stages. The samples were collected from 9:00 to 10:00 a.m. for each treatment. Here, four leaves were collected, placed in self-sealing bags, marked, and then quickly placed in a refrigerator. The 80% acetone extraction method was used to determine the contents of various pigments. The absorbance of the extraction solution was measured at 663, 645, and 470 nm, and the contents of chlorophylls a and b and carotenoids in leaves per unit mass were calculated according to the Arnon correction method^37^.

#### Determination of leaf fluorescence parameters

Leaf fluorescence parameters were measured by a PAM-2500 portable modulated chlorophyll fluorescence meter at the booting (23 July), heading (11 August), and maturity (13 September) stages. Leaves were selected using the protocol adopted for photosynthetic pigment content determination. The initial fluorescence (F_o_) and maximum fluorescence (F_m_) under dark adaptation were measured, then, the maximal quantum yield of PSII (F_v_/F_m_) was calculated using the formula (F_m_ − F_o_)/F_m_. Measurement of the minimum fluorescence (F_o_′), maximum fluorescence (F_m_′), and real-time fluorescence yield (F) under light adaptation revealed the actual quantum yield of PSII (Φ_PSII_), photochemical quenching coefficient (qP), non-photochemical quenching coefficient (qN), quantum yield of non-regulatory energy dissipation at PSII(Y(NO)), quantum yield of regulatory energy dissipation at PSII (Y(NPQ)), and relative electron transfer rate at PSII (ETR).

#### Measurement of yield and its components

At the maturity stage, rice yield and its related components were determined by analyzing 90 hills in each subplot and adjusted to a moisture content of 0.135 g H_2_O g^−1^ fresh weight. Six representative hills were selected on the basis of the average number of stems and tillers in the field survey as test samples to investigate the yield components of rice.

#### Statistical analysis

The data were analyzed by SAS9.0 (SAS Institute, Cary, NC, USA).

## Data Availability

All data generated or analysed during this study are included in the article.
